# Comparative analysis of neutralization assays performed using live SARS-CoV-2 virus and pseudovirus to assess immunogenicity of a bivalent SARS-CoV-2 protein vaccine in humans

**DOI:** 10.3389/fimmu.2025.1650083

**Published:** 2025-09-23

**Authors:** Cuige Gao, Jiang Yi, Adam Baidoo, Dongfang Liu, Jing Li, Dongyang Li, Jian Li, Qiang Zhou, Liangzhi Xie

**Affiliations:** ^1^ Beijing Engineering Research Center of Protein and Antibody, Sinocelltech Ltd, Beijing, China; ^2^ Beijing Key Laboratory of Monoclonal Antibody Research and Development, Sino Biological Inc., Beijing, China; ^3^ Cell Culture Engineering Center, Chinese Academy of Medical Sciences & Peking Union Medical College, Beijing, China

**Keywords:** SARS-CoV-2, immunogenicity, neutralization assays, vaccination, COVID-19

## Abstract

**Objectives:**

The rapid emergence of SARS-CoV-2 prompted accelerated vaccine development, with neutralization assays serving as essential tools to evaluate vaccine-induced immune responses.

**Methods:**

A *post-hoc* analysis of a Phase I/II trial evaluated the immunogenicity of a bivalent SARS-CoV-2 protein vaccine. We assessed vaccine immunogenicity using live virus neutralization assays (LVNA) and pseudotyped virus neutralization assays (PVNA) to measure antibody responses against different variants, including Alpha B.1.1.7, Beta B.1.351, and Delta B.1.617.2. Various statistical techniques, including correlation coefficients, regression models, and Bland–Altman plots, were employed to assess the relationship between antibody titers from the two assays.

**Results:**

We analyzed 324 samples for Alpha and Beta variants and 505 for Delta. Compared with LVNA, the sensitivity and specificity of PVNA were over 90% across all variants, with accuracy rates of 98.8% for Alpha, 99.1% for Beta, and 94.3% for Delta. The Pearson correlation between PVNA and LVNA was strong for Alpha (CORR = 0.9614), Beta (CORR = 0.9517), and Delta (CORR = 0.9072). Bland-Altman plots and Kernel density plots indicated good agreement between PVNA and LVNA.

**Conclusions:**

Our findings demonstrate a strong correlation between PVNA and LVNA results, supporting PVNA as a safe, scalable, and reliable surrogate for LVNA in evaluating vaccine immunogenicity.

## 1 Introduction

The emergence of Severe Acute Respiratory Syndrome Coronavirus 2 (SARS-CoV-2) necessitated the rapid development and deployment of vaccines and therapeutic agents. A critical component in evaluating the effectiveness of these interventions is the neutralization assay, which quantifies the ability of antibodies to inhibit virus infection. By measuring the neutralizing activity, researchers can determine the magnitude and durability of immune responses elicited by the vaccine candidates, guiding modifications and improvements in their formulation (1, 2). Furthermore, understanding variations in neutralization efficacy against emerging viral variants is essential for demonstrating cross-reactive neutralizing activity and ensuring long-term protection against evolving strains (3–6).

Traditional neutralization assays using live viruses directly measure antibody effectiveness against authentic pathogens and remain the gold standard for evaluating neutralizing antibodies (7, 8). However, live virus neutralization assays (LVNAs) pose several challenges, such as biosafety issues and the requirement for high-level containment facilities. To overcome these limitations, pseudotyped virus neutralization assays (PVNAs) employ non-pathogenic viruses engineered to express the SARS-CoV-2 spike protein, enabling a safe and reliable simulation of viral entry. Core viruses like vesicular stomatitis virus (VSV) and lentiviruses (e.g., HIV-1) are commonly used, making PVNAs suitable for biosafety level-2 (BSL-2) laboratories, in contrast to LVNA, which typically require BSL-3 containment (4, 9). Additionally, PVNAs offer high-throughput capacity and adaptability to various viral strains, making them particularly valuable for research on evolving pathogens.

However, inherent methodological differences between PVNAs and LVNAs—such as variations in viral life cycle stages, entry mechanisms, and cell tropism—may lead to discrepancies in neutralization measurements. While PVNAs serve as valuable tools for initial screening and mechanistic studies, they should be validated with LVNAs to ensure biological relevance to natural infections. Understanding the correlation between the two is crucial for validating PVNAs as reliable proxies for LVNAs in vaccine and therapeutic development. Previous studies have reported varying degrees of concordance between PVNAs and LVNAs (8, 10–15). Most of these investigations, however, were based on small sample sizes, and utilized sera from COVID-19 convalescents rather than vaccinated individuals. Additionally, only a limited number of them evaluated the correlation across multiple SARS-CoV-2 variants (8, 14, 15). One study compared the serum neutralizing activities in participants who received COVID-19 vaccines with those who had experienced breakthrough infections of different SARS-CoV-2 variants (16). The results showed that vaccination induced higher and broader neutralizing antibody titers against various variants compared with breakthrough infection.

Here, we intended to access the correlation between neutralizing antibodies against different variants measured by PVNA and LVNA among vaccinated individuals based on a larger sample size. This is a *post-hoc* analysis of a phase I/II clinical trial that evaluated a bivalent protein-based COVID-19 vaccine, providing insights into the relative performance and reliability of PVNA and LVNA.

## 2 Materials and methods

### 2.1 Study design and participants

This comprehensive *post-hoc* analysis examined data from a Phase I/II clinical trial of vaccine interventions. Phase I assessed the safety of different dose levels of a vaccine in healthy volunteers, while Phase II evaluated its immunogenicity in a larger, diverse population, also monitoring safety. Participants who had neither been infected with SARS-CoV-2 nor received any SARS-CoV-2 vaccines were included. A total of 476 participants were enrolled, with 84 assigned to Phase I and 392 to Phase II. They were all randomized to receive two doses of study vaccinations, including SCTV01C, SCT-VA02B, or normal saline, 28 days apart, as the primary series of vaccination. SCTV01C is a recombinant bivalent vaccine comprised of the trimeric spike extracellular domain (S-ECD) of SARS-CoV-2 variants Alpha and Beta, and adjuvanted with SCT-VA02B, a squalene-based oil-in-water emulsion. Two doses of SCTV01C were used in this study, which were 20 μg and 40 μg. Immunogenicity was evaluated both pre- and post-vaccination by detecting the titers of neutralizing antibodies. The primary results of this clinical trial have been published (17, 18). This analysis aimed to uncover correlations between neutralizing antibody results from LVNA and PVNA across different SARS-CoV-2 variants. Sera of those vaccinated with SCTV01C were selected for analysis.

The Ethics Committee of Beijing Center for Disease Control and Prevention and the National Medical Products Administration of China approved the protocol (NCT05148091). The study adhered to the Good Clinical Practice and the Declaration of Helsinki. All enrolled participants signed the informed consent.

### 2.2 Neutralization assays

Neutralizing antibodies against SARS-CoV-2 Alpha B.1.1.7, Beta B.1.351, and Delta B.1.617.2 variants were measured using LVNA and PVNA.

#### 2.2.1 SARS-CoV-2 microneutralization assay

The LVNA used in this study was the microneutralization assay (MNA) and has been described previously (17). The sera were heat inactivated at 56°C for 30 minutes, and then serially diluted two-fold starting at 1:8. All serial dilutions of test samples were prepared in duplicate in a separate dilution plate. The 50 μl diluted sera were mixed with an equal volume of SARS-CoV-2 variants (1000 TCID50 per well of Alpha B.1.1.7, Beta B.1.351, or Delta B.1.617.2 variants, respectively) and incubated for 1~2 hours at 37°C, 5% CO2. The virus/serum mixtures were then transferred to sub-confluent Vero E6 cell monolayer plates (E6 cells were pre-seeded 24 hours beforehand). Plates were incubated for 3–5 days at 37°C, 5% CO2. The residual non-neutralized virus was detected via cytopathic effect (CPE) by microscopic scoring. The microneutralization titers (MN50) were defined as the reciprocal of the highest dilution that protected 50% of wells from cytopathy and were calculated using the Reed-Muench method equation.

#### 2.2.2 SARS-CoV-2 pseudotyped virus neutralization assay

The establishment and validation of the SARS-CoV-2 pseudotyped virus neutralization assay (PVNA) have been reported (9, 19). A pseudovirus containing the luciferase gene was produced using a VSV pseudovirus production system. The sera were diluted serially three-fold starting at 1:30 to a final dilution of 1:7290. The diluted serum was mixed with 50 μl pseudovirus (1000 TCID50/well) and incubated with Huh7 cells (2 × 10^4^ cells/well). The neutralizing antibodies in sera can block the pseudovirus entry into target cells. Pseudoviruses that successfully enter cells can express luciferase. The luminescence signal, measured as relative luminescence units (RLU) of the inoculum after cell lysis with substrate addition using a microplate luminometer, indicates infection levels. The amount of neutralized pseudovirus was determined by the reduction of RLU relative to the virus control wells (cells infected with pseudovirus without serum, set as 100% infection). Cell-only wells served as background controls. The half maximal effective concentration (EC50) titer of a serum sample was defined as the reciprocal of the dilution that neutralized 50% of the pseudovirus and was calculated using the Reed-Muench equation.

### 2.3 Statistical analysis

Among all the sera (before or after vaccination) from the analysis population, those with both LVNA and PVNA test results of the same SARS-CoV-2 variant (Alpha, Beta, or Delta) were selected for analysis. The results of the three SARS-CoV-2 variants were analyzed separately. The statistical analyses were conducted using SAS software (version 9.4). The initial dilution fold was established as the lower limit of quantitation (LLOQ). The LLOQ was 8 for the SARS-CoV-2 Microneutralization Assay and 30 for the SARS-CoV-2 Pseudotyped Virus Neutralization Assay. Any serological values below the LLOQ were set to 0.5 times LLOQ.

In the analysis of diagnostic performance (error matrix), antibody titers against SARS-CoV-2 equal to or greater than LLOQ were labeled as “Positive,” while those below LLOQ were labeled as “Negative.” The antibody titers of LVNA were taken as the reference (“true”) results. When compared with the antibody titers of PVNA, the following four classification outcomes are obtained: 1) True Positive (TP): Both LVNA and PVNA are positive. 2) False Positive (FP): LVNA is negative, but PVNA is positive. 3) True Negative (TN): Both LVNA and PVNA are negative. 4) False Negative (FN): LVNA is positive, but PVNA is negative. Specificity is defined as TN/(TN+FP), sensitivity is defined as TP/(TP+FN), and accuracy is defined as (TN+TP)/(TN+TP+FN+FP). Misclassifications were counted and displayed in an error matrix table. The Pearson correlation coefficient and a linear regression model were used to measure the strength of the relationship between PVNA and LVNA. All antibody titers were log-transformed to the base 10 before calculation.

To evaluate the agreement between PVNA and LVNA, a Bland–Altman plot (20) and a Kernel density plot were plotted. The Bland–Altman plot is a scatter plot of the mean PVNA and LVNA at each measurement point, along with their differences. The PVNA and LVNA were log-transformed to the base 10 before calculating the mean and difference. The 95% limits of agreement (LOAs) and the maximum acceptable difference (MAD) were calculated. The LOAs were constructed as a V-shaped limit (21), and the MAD was set to 0.5 times the titer measured by PVNA. If the observed PVNA-LVNA difference is below the MAD value, it is considered that the difference has no significant biological effect. The Gaussian kernel is chosen to plot the kernel density plot, which describes the probability distribution of the fold increase relative to the baseline of antibody titers after vaccination. The fold increase was log-transformed to base 10 when plotted.

## 3 Results

### 3.1 The neutralizing antibody titers

Descriptive analysis was performed on all the sera, categorized by the sampling visit times, assay methods, and corresponding variants. The Geometric mean titers (GMTs) and their 95% confidence intervals were calculated for each category (**Table 1**). At some time points, neutralizing antibodies against the Delta variant were not measured because the variant emerged later. It can be seen that at baseline, almost all the antibody titers were below the LLOQ. At 14 days after the second vaccination, the antibody titers increased significantly compared to baseline, followed by a gradual decrease over time. However, the neutralizing antibodies increased significantly again on 365 days after vaccination. The possible reasons might include known or unknown SARS-CoV-2 infection and close contact with COVID-19 individuals during the SARS-CoV-2 epidemic due to the decline of neutralizing antibodies and the emergence of new variants.

**Table 1:** Number of sera and geometric mean (95% CI) of neutralizing antibody titers.

Time point	Alpha			Beta			Delta		
	N	LVNA	PVNA	N	LVNA	PVNA	N	LVNA	PVNA
Baseline	60	4 (NA, NA)	15 (15, 16)	60	4 (NA, NA)	15 (15, 16)	296	4 (NA, NA)	17 (16, 18)
14-days after 2^nd^ vaccination	56	1083 (902, 1300)	3528 (2922, 4259)	56	1018 (805, 1287)	3004 (2456, 3673)	0	–	–
28-days after 2^nd^ vaccination	56	775 (625, 961)	2376 (1918, 2942)	56	525 (393, 702)	2068 (1622, 2637)	209	138 (118, 162)	297 (260, 339)
90-days after 2^nd^ vaccination	53	220 (161, 301)	580 (442, 762)	53	196 (143, 268)	526 (399, 694)	0	–	–
180-days after 2^nd^ vaccination	49	110 (73, 164)	312 (222, 441)	49	133 (93, 189)	227 (163, 316)	0	–	–
365-days after 2^nd^ vaccination	50	3126 (2204, 4433)	5856 (4151, 8262)	50	2135 (1461, 3120)	4483 (3104, 6474)	0	–	–

### 3.2 The diagnostic performance

The number of sera tested for the Alpha, Beta, and Delta variants was 324, 324, and 505, respectively, with the results shown in **Table 2**. The data indicate that PVNA exhibits excellent sensitivity relative to LVNA across all variants tested. In detecting all three variants, only one LVNA-positive sample was negative in the PVNA test for the Delta variant. The specificity of PVNA for LVNA was also good, with a specificity greater than 90% for all three variants. The accuracy of PVNA for LVNA was 98.8%, 99.1%, and 94.3% for the Alpha, Beta, and Delta variants, respectively, reflecting the high consistency in the test results.

**Table 2:** Error matrix of PVNA and LVNA.

Variants	PVNA	LVNA			Diagnostic Performance		
Alpha		Negative	Positive	Total	Specificity	Sensitivity	Accuracy
	Negative	60	0	60	93.8% (84.8%, 98.3%)	100% (98.6%, 100%)	98.8% (96.9%, 99.7%)
	Positive	4	260	264			
	Total	64	260	324			
Beta		Negative	Positive	Total	Specificity	Sensitivity	Accuracy
	Negative	60	0	60	95.2% (86.7%, 99.0%)	100% (98.6%, 100%)	99.1% (97.3%, 99.8%)
	Positive	3	261	264			
	Total	63	261	324			
Delta		Negative	Positive	Total	Specificity	Sensitivity	Accuracy
	Negative	272	1	273	90.7% (86.8%, 93.7%)	99.5% (97.3%, 99.99%)	94.3% (91.9%, 96.1%)
	Positive	28	204	232			
	Total	300	205	505			

### 3.3 The correlation between PVNA and LVNA

**Figure 1:**
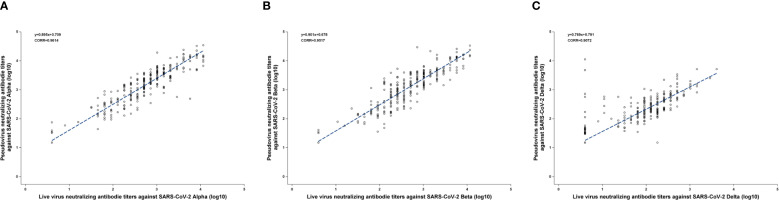
The Pearson correlation coefficients between PVNA and LVNA. **(A)** Alpha variant. **(B)** Beta variant. **(C)** Delta variant. The blue line was constructed by the linear equation from the upper left corner of the figure. The Pearson correlation coefficient was represented as CORR in the figure.

The Pearson correlation coefficients (CORR) between PVNA and LVNA were 0.9614, 0.9517, and 0.9072 for the Alpha, Beta, and Delta variants, respectively, indicating that PVNA and LVNA have a strong positive correlation. Almost all the points are distributed near the regression line, indicating a strong linear relationship between PVNA and LVNA for Alpha and Beta variants (**Figures 1A, B**). For the Delta variant, although most points were distributed near the regression line, there were some points where the titers of LVNA were below the LLOQ, while those of PVNA remained high. This resulted in a weaker correlation between LVNA and PVNA for the Delta variant compared to the Alpha and Beta variants.

### 3.4 Bland-Altman analysis

**Figure 2:**
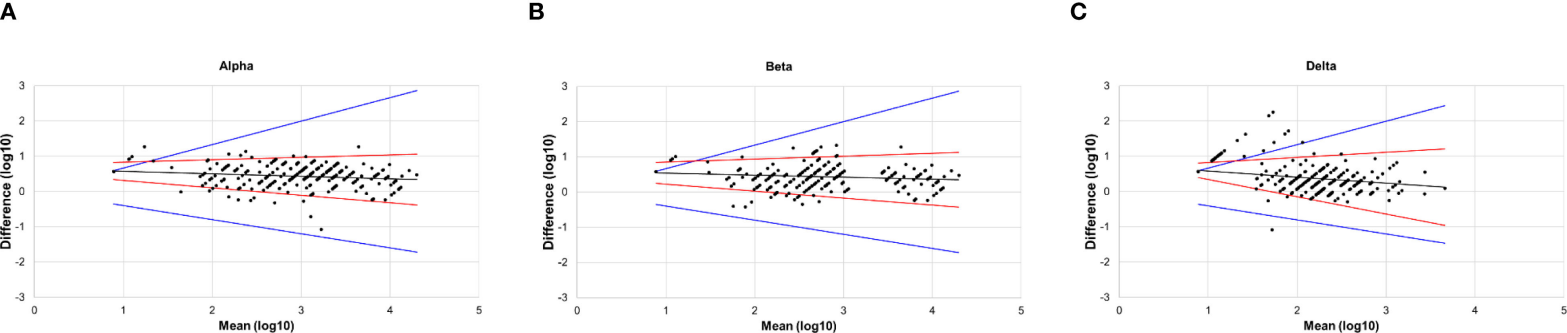
The Bland–Altman plot of PVNA and LVNA. **(A)** Alpha variant. **(B)** Beta variant. **(C)** Delta variant. The x-axis represented the mean of the log-transformed titers of PVNA and LVNA, while the y-axis represented the difference between the log-transformed titers of PVNA and LVNA. The black lines were the regression line, the red lines were the 95% limits of agreement, and the blue lines indicated the maximum acceptable difference.

The results of the Bland–Altman analysis are shown in **Figure 2**. Almost all the points in the figures were distributed close to the regression line, and the difference did not increase as the mean increased. Additionally, the angle between the two LOA lines was very small, indicating good agreement between PVNA and LVNA. For Alpha and Beta variants, only a few points lay outside the range of the MAD, with very few outliers (**Figures 2A, B**). However, for the Delta variant, several points appeared above the MAD (**Figure 2C**). These points correspond to the sera where titers of LVNA were below the LLOQ, but those of PVNA still had a detectable value. Aside from these points, the rest showed high agreement between PVNA and LVNA.

### 3.5 The probability distribution of the fold increase relative to the baseline of PVNA and LVNA

**Figure 3:**
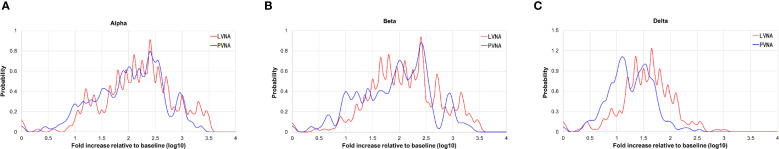
The Kernel density plot of the titer fold increases from the baseline after vaccination. **(A)** Alpha variant. **(B)** Beta variant. **(C)** Delta variant.

As the GMT of PVNA was higher than that of LVNA, the distribution of fold increase relative to baseline was used to analyze the agreement between PVNA and LVNA. For Alpha and Beta variants, the distribution curves of PVNA results overlap almost entirely with those of LVNA, indicating good agreement between these two assays (**Figures 3A, B**). For the Delta variant, the curves of PVNA and LVNA were similar in shape but did not overlap, with the relative baseline fold increase of PVNA being slightly lower than that of LVNA (**Figure 3C**). The agreement of fold increase relative to baseline between PVNA and LVNA for the Delta variant was slightly lower than that for the Alpha and Beta variants.

## 4 Discussion

Antibody detection and quantification methods play a crucial role in assessing immune response post-infection or vaccination. Although LVNA directly evaluates neutralizing capability in an infectious context, its application is constrained by several challenges, including stringent safety protocols, regulatory constraints, and variability in viral strain availability (22). These limitations hinder the scale and reproducibility of experimental studies, ultimately affecting the efficiency of vaccine and therapeutic development efforts (23). In contrast, PVNA demonstrates notable advantages, including enhanced safety, broader accessibility, and versatility in testing for various viral threats, such as SARS-CoV-2 (24), HIV (25), HPV (26), Influenza (27), and others. These characteristics illustrate the growing preference for PVNA in virology research, providing a vital tool for advancing the study of viral infections and immune responses (28, 29). Notably, PVNA has been recognized as an acceptable assay for assessing immunogenicity endpoints in the FDA guidance for COVID-19 vaccine development (30).

Understanding the correlation between LVNA and PVNA is important for determining whether pseudotyped-based assays can effectively replace live virus assays in viral testing. This knowledge ensures both the practicality of testing methods and the safety involved, given the significant risks associated with handling live viruses. Our findings indicate a strong overall agreement between these two methods, while also highlighting essential nuances that researchers should consider in vaccine development and therapeutic evaluations.

We employed various statistical methods to analyze the correlation between LVNA and PVNA. Diagnostic Performance shows a high level of agreement in qualitative results between PVNA and LVNA, while correlation and regression analyses confirm consistency in quantitative results. Bland–Altman analysis aims to determine if there are systematic differences between PVNA and LVNA. The consistency observed across these different approaches provides a robust foundation for understanding the relationship between the two methodologies. The analysis of diagnostic performance between PVNA and LVNA demonstrated that the sensitivity and specificity of PVNA exceeded 90% for all variants compared with LVNA, indicating its potential as a robust and reliable tool for assessing neutralizing antibody responses in populations exposed to SARS-CoV-2. Furthermore, the accuracy rates recorded were 98.8% for Alpha, 99.1% for Beta, and 94.3% for Delta. These results underscored the effectiveness of PVNA in distinguishing neutralization capabilities against these variants. The Pearson correlation coefficients of 0.9614 for Alpha and 0.9517 for Beta demonstrated that PVNA could effectively replicate the neutralization dynamics of live viruses in controlled settings. Conversely, the correlation for Delta (CORR = 0.9072) was slightly weaker, suggesting potential variability in neutralization capabilities, which warrants further investigation into the impact of specific mutations in this variant. SCTV01C is a bivalent vaccine made from S-ECD proteins of SARS-CoV-2 variants Alpha and Beta. The neutralizing antibody titer against Delta was lower than that against Alpha and Beta, possibly because of a mismatch between the vaccine-contained antigens and the Delta variant. Bland-Altman analysis further supports the reliability of PVNA, showing good agreement with LVNA results. We visualized any systematic bias and identified outliers by plotting the difference between LVNA and PVNA results against their average. Our analysis revealed minimal bias and narrow limits of agreement, indicating that the discrepancies between the two methods are generally small and random. Notably, while the geometric mean titer (GMT) of PVNA was higher than that of LVNA for the Delta variant, the slightly lower fold increase in titers of PVNA may indicate that certain mutations in the spike protein of Delta could reduce the efficacy of neutralization. Future research should continue to refine these methodologies to enhance their predictive accuracy and broaden their applicability in vaccine development and evaluation.

It is important to interpret the correlation between PVNA and LVNA carefully. While our findings support PVNA’s reliability, differences in methodology - such as the use of engineered particles versus live viruses - can affect neutralization effectiveness. Additionally, variations in the quality of viruses used in these assays may also cause differences between the two tests. Dead and ghost virus particles that bind to antibodies will reduce the antibody titers in LVNA, which could explain why some samples show negative results in the LVNA method but have low antibody titers in other tests PVNA. These variations may be due to differing biological testing contexts and immune responses. PVNA utilizes recombinant, replication-deficient viruses engineered to express SARS-CoV-2 spike proteins on their surface. The ability of antibodies to neutralize viruses may not fully translate from pseudotyped to live viruses due to the complex interactions present in live viral infections (8, 13, 31).

This study has several limitations. Notably, neutralizing antibody responses against the Omicron variant were not assessed, leaving the impact of its mutations on assay correlation unexplored. Additionally, conducting LVNA and PVNA assays by different experimenters may have introduced procedural variability.

## 5 Conclusion

In conclusion, while LVNA provides a precise measure of vaccine-induced immunity, PVNA offers benefits in safety, cost, and scalability. It is essential to comprehend the strengths and limitations of both methods for effective vaccine evaluation, particularly in light of emerging SARS-CoV-2 variants. Future research should aim to enhance PVNA techniques to represent natural infections better and improve accuracy for various viral strains. By combining efforts, both approaches can accelerate the development of effective vaccines against evolving pathogens.

## Data Availability

The raw data supporting the conclusions of this article will be made available by the authors, without undue reservation.
